# Addressing multicollinearity in general linear model: A novel approach for ridge parameter with performance comparison

**DOI:** 10.1371/journal.pone.0335072

**Published:** 2025-10-24

**Authors:** Muhammad Luqman, Sajjad Haider Bhatti, Demet Aydin, Mohsin Jamil

**Affiliations:** 1 College of Statistical Sciences, University of the Punjab, Lahore, Pakistan; 2 Data Science and Analytics, Sinop University, Sinop, Türkiye; University of Sargodha, PAKISTAN

## Abstract

The problem of ill-conditioned data or multicollinearity is common in regression modelling. The problem results in imprecise parameter estimation which leads to inability of gauging true impact of explanatory variables on the response. Also, due to strong multicollinearity, standard errors of parameter estimates get inflated leading to wider confidence intervals and hence increased risk of type-II error. To handle the problem, different approaches have been proposed in literature. Primarily, such techniques penalize the coefficient estimates in one way or other. Ridge regression is one of the most applied among such techniques. In ridge regression, a penalty term is added in the objective function of the general linear model. That penalty term introduces a small amount of bias in parameter estimates with an objective to decrease the mean square error. In the current article, some new choices for ridge constant are proposed. The performance of proposed ridge choices are compared through Monte Carlo simulations under different scenarios, using mean square error as measure of performance. The simulation results indicate that the proposed ridge estimator performs better than existing ridge constants, in most cases catering for severity of multicollinearity, number of explanatory variables, sample size and error variance structure. The simulation results were further corroborated by comparing performance of proposed ridge penalties using two real-life applications.

## 1. Introduction

In multiple regression, it is difficult to interpret the coefficient estimates if different explanatory variables are highly correlated among each other. Moreover, strong multicollinearity leads to inflated standard errors of parameters estimates which further result in wider confidence intervals and increased risk of type-II error [1–3].

Consider the multiple linear regression model in matrix form,


Y=Xβ+μ (1)

where Y is a (n×1) vector of response variable, X is (n×r) matrix with p explanatory variables and a constant term (i.er=p+1), β is (r×1) vector of parameters with an intercept and p slope coefficients and μ is (n×1) vector of random error term.

In regression analysis, a fundamental assumption is the absence of strong linear dependence among explanatory variables. Termed as no-multicollinearity, this assumption means that no pair or group of independent variables have a strong linear relationship [2]. Under common assumptions [3,4] of general linear model including absence of strong linear dependence (multicollinearity) among explanatory variables, the parameter vector β can be estimated through ordinary least squares approach as,


$${\hat{\beta }}_{OLS}={\left(X^{\prime }X\right)}^{-1}X^{\prime }Y$$ (2)

OR


$${\hat{\beta }}_{OLS}=min\left\{{\sum_{j=1}^{n}\left(Y_{j}-\beta_{0}-\sum_{i=1}^{p}\beta_{i}X_{ji}\right)}^{2}\right\}\cdot$$ (3)

However, in many practical situations, the assumption of no-multicollinearity is not valid and explanatory variables have strong correlation among each other. In such situations, it is challenging to accurately estimate model parameters using the least squares method given in Equation (2).

To deal with the problem of strong multicollinearity or ill-conditioned data, Hoerl and Kennard [5] pioneered the idea of ridge regression (RR) as an alternative way to estimate the parameters of regression model. To reduce the adverse effects of multicollinearity, the idea of RR is to penalize the large coefficients through a regularization constant which results in improved stability of estimates. Simply, a small amount of bias is introduced that results in lower variance of the parameter estimates. The RR estimates can be obtained as,


$${\hat{\beta }}_{RR}={\left(X^{\prime }X+kI_{p}\right)}^{-1}X^{\prime }Y$$ (4)

OR


$${\hat{\beta }}_{RR}=min\left\{{\sum_{j=1}^{n}\left(Y_{j}-\sum_{i=1}^{p}\beta_{i}X_{ji}\right)}^{2}+k\sum_{i=1}^{p}\beta_{i}^{2}\right\}$$ (5)

where k>0 is ridge parameter and $I_{p}$ is the identity matrix of order p representing number of regressors. It is common practice in penalized regression approaches to remove the constant term from X matrix and then estimate it after estimating the p slope coefficients. With k=0, the RR reduces to common OLS estimation. It is the ridge constant k which controls the amount of bias in coefficient estimates and it is generally chosen with an objective to achieve the lower mean square error (MSE) of the elements of the estimated parameter vector $\hat{\beta }$. The performance of any RR estimator mainly depends on the choice of ridge penalty. Since the emergence of idea, many approaches have been proposed in literature to choose the ridge parameter [1,5–19]. Each of these choices offers a different approach to determine the optimal value for ridge parameter (k) and has been shown to work better in different scenarios encountered in practical applications.

Later, this idea lead to other classes of biased estimation methods like liu, lasso and elastic-net approaches [9,16–18,20,21]. These estimation strategies penalize the coefficients in ways different from RR.

Although regularization techniques have been extensively explored in literature, but we believe there exist ample space for more refined, data driven (incorporating severity of problem, i.e., multicollinearity) penalization strategies, especially in applied settings where usual/common assumptions may not be valid every time. Motivated with the objective of proposing ridge estimators related to the underlying level of multicollinearity in the data at hand, in the current article, we propose two new penalty terms to be used in ridge regression and compare their performance with some existing historical and recent choices for ridge parameter.

The rest of the article is structured as: Section 2 provides a brief overview of existing ridge penalties to be compared and new choices for ridge penalty being proposed in this study. The section also explains the simulation scheme. Section 3 presents results from Monte Carlo simulations obtained under different scenarios. Section 4 provides the results obtained by comparing existing and proposed approaches on two real-life datasets. Finally, Section 5 concludes the article.

## 2. Methodology

Consider the general linear model given in Equation (1) as,


Y=Xβ+μ

$$Y_{j}=\beta_{0}+\beta_{1}X_{j1}+\beta_{2}X_{j2}+\cdots +\beta_{p}X_{jp}+\mu_{j},\;j=1,2,3,\cdots ,n$$ (6)

$$Y_{j}=\beta_{0}+\sum_{j=1}^{n}\sum_{i=1}^{p}\beta_{i}X_{ji}+\mu_{j}$$ (7)

The general linear model given in Equation (1) can be represented in canonical form as,


y=Zα+μ (8)

where $Z=X^{*}E,$ $X^{*}$ is the matrix of standardized explanatory variables, E is an orthogonal matrix such that $E^{\prime }E=I_{p}$ and $E^{\prime }X^{{*}^{\prime }}X^{*}E=\Lambda$, where $\Lambda =diag(\lambda_{\text{1}},\lambda_{\text{2}},\ldots ,\lambda_{p})$ is the diagonal matrix consisting of the eigen values of $X^{{*}^{\prime }}X^{*}$, and y is response variable centered to its mean. Then the OLS and RR estimators of α in canonical form are given as;


$${\hat{\alpha }}_{OLS}={\left(Z^{\prime }Z\right)}^{-1}Z^{\prime }y$$ (9)

$${\hat{\alpha }}_{RR}={\left(Z^{\prime }Z+kI_{p}\right)}^{-1}Z^{\prime }y$$ (10)

where ,k>0, is the ridge parameter. Once $\hat{\alpha }$ are computed, standardized coefficients (${\hat{\beta }}_{scaled}$) and parameter estimates on original units ($\hat{\beta })$ can be obtained as:


$${\hat{\beta }}_{scaled}=E\hat{\alpha }$$ (11)

$$\hat{\beta }={\hat{\beta }}_{scaled}*\frac{\text{1}}{\sqrt{diag(cov(X))}}$$ (12)

and


$${\hat{\beta }}_{0}=\bar{Y}-\sum_{i=1}^{p}{\hat{\beta }}_{i}{\bar{X}}_{i}$$ (13)

The mean square error of OLS and RR estimators can be computed respectively as,


$$MSE\left({\hat{\alpha }}_{OLS}\right)={\hat{\sigma }}^{2}\sum_{i=1}^{p}\frac{\text{1}}{\lambda_{i}}$$ (14)

$$MSE\left({\hat{\alpha }}_{RR}\right)={\hat{\sigma }}^{2}\sum_{i=1}^{p}\left(\frac{\lambda_{i}}{{\left(\lambda_{i}+k\right)}^{2}}\right)+k^{2}\sum_{i=1}^{p}\left(\frac{{\hat{\alpha }}_{i}^{2}}{{\left(\lambda_{i}+k\right)}^{2}}\right)$$ (15)

where


$${\hat{\sigma }}^{2}=\frac{\sum_{j=1}^{n}{\hat{\mu }}_{j}^{2}}{n-p}\;\;\;with\;\;\;\hat{\mu }=y-\hat{y}\;\;\;and\;\;\;\hat{y}=X^{*}\hat{\alpha }.$$ (16)

### 2.1 Existing ridge penalties

As already discussed, ridge regression was pioneered by Hoerl and Kennard [5] as an alternative method to ordinary least squares (OLS) for dealing ill-conditioned data. After their pioneering work, numerous ridge parameters have been suggested by different authors in literature focused on dealing with multicollinear data. The ridge choices which have been used in current study for comparison are briefly described in following sub-sections.

#### 2.1.1 Hoerl and Kennard (1970).

In their initial work, Hoerl and Kennard [5] proposed the following ridge constant to be used in ridge regression,


$$k_{HK}=\frac{{\hat{\sigma }}^{2}}{{\hat{\alpha }}_{\max}^{2}}$$ (17)

Later on, several researchers proposed estimators that incorporated various forms of the Hoerl and Kennard [5] estimator.

#### 2.1.2 Hoerl, Kennard and Baldwin (1975).

In another work, Hoerl et al. [22] recommended a different ridge parameter. It is actually based on the mean of estimates from canonical form instead of just taking the maximum of these estimates. Their proposed ridge penalty is given as,


$$k_{HKB}=\frac{p{\hat{\sigma }}^{2}}{\sum_{i=1}^{p}{\hat{\alpha }}_{i}^{2}}$$ (18)

#### 2.1.3 Lawless and Wang (1976).

Lawless and Wang [12] suggested an estimator by substituting the denominator of $k_{HKB}$ with a weighted sum of $\hat{\alpha }$ taking corresponding eigen values $\left(\lambda_{i}\right)$ as weights. Their proposed estimator is given as,


$$k_{LW}=\frac{p{\hat{\sigma }}^{2}}{\sum_{i=1}^{p}\lambda_{i}{\hat{\alpha }}_{i}^{2}}$$ (19)

#### 2.1.4 Hocking, Speed and Lynn (1976).

An improved version of the estimator suggested by Lawless and Wang [12] was proposed by Hocking et al. [23] as,


$$k_{HSL}=\frac{{\hat{\sigma }}^{2}\sum_{i=1}^{p}{\left(\lambda_{i}{\hat{\alpha }}_{i}^{2}\right)}^{2}}{{\left(\sum_{i=1}^{p}\lambda_{i}{\hat{\alpha }}_{i}^{2}\right)}^{2}}$$ (20)

#### 2.1.5 Kibria (2003).

Kibria [24] proposed three estimators based on arithmetic mean, geometric mean, and median of the term ${\hat{\sigma }}^{\text{2}}/{\hat{\alpha }}_{i}^{\text{2}}$ (instead of taking maximum as in Hoerl and Kennard, 1970). The three proposed ridge estimators are defined as,


$$k_{KAM}=\frac{{\hat{\sigma }}^{2}}{p}\sum_{i=1}^{p}\frac{\text{1}}{{\hat{\alpha }}_{i}^{2}}$$ (21)

$$k_{KGM}={\hat{\sigma }}^{2}{\left(\prod_{i=1}^{p}\frac{\text{1}}{{\hat{\alpha }}_{i}^{2}}\right)}^{\frac{\text{1}}{p}}$$ (22)

$$k_{KMED}=median\left(\frac{{\hat{\sigma }}^{2}}{{\hat{\alpha }}_{i}^{2}}\right)$$ (23)

#### 2.1.6 Khalaf and Shukur (2005).

Khalaf and Shukur [25] proposed a ridge estimator which is based on the estimate of error variance, maximum eigen value, and regression coefficients in canonical form as follows:


$$k_{KS}=\frac{{\hat{\sigma }}^{2}\lambda_{\max}}{(n-p){\hat{\sigma }}^{2}+\lambda_{\max}{\hat{\alpha }}_{\max}^{2}}$$ (24)

#### 2.1.7 Khalaf, Mansson and Shukur (2013).

Khalaf et al. [15] proposed some modifications in different ridge estimators by multiplying with a certain weight. In the present study, for comparison, we have taken one ridge estimator from Khalaf et al. [15] which is given as,


$$k_{KMS}=\frac{\lambda_{\max}}{\sum_{i=1}^{p}\left|{\hat{\alpha }}_{i}\right|}\left(\frac{{\hat{\sigma }}^{2}}{{\hat{\alpha }}_{\max}^{2}}\right)$$ (25)

#### 2.1.8 Karaibrahimoğlu, Asar and Genç (2016).

Karaibrahimoğlu et al. [26] suggested a new choice for ridge parameter by adjusting the form proposed by Dorugade [27]. Their suggested ridge parameter is given as,


$$k_{KAG}=\frac{\sqrt{5}*{\hat{\sigma }}^{2}}{\lambda_{\max}\sum_{i=1}^{p}{\hat{\alpha }}_{i}^{2}}$$ (26)

#### 2.1.9 Shabbir, Chand and Iqbal (2024).

Recently, Shabbir et al. [28] proposed a ridge parameter which was found superior than some other ridge parameters in simulation studies. The mentioned estimator is defined as,


$$k_{SCI}=p^{\left(1+\frac{\text{1}}{p}\right)}\sqrt{{\hat{\sigma }}^{2}}$$ (27)

### 2.2 Proposed ridge estimators

The core motivation behind our proposed ridge parameters is that they are data-driven, as they are function of the condition number which is considered a meaningful measure of severity of multicollinearity. So, the amount of shrinkage by our proposed ridge constants is related to the underlying level of multicollinearity in the data at hand. While many choices for ridge parameter exist in empirical literature, but most of them assume a globally fixed penalty term. The ridge estimators that take the level of multicollinearity in explanatory variables in the data into account remain relatively scarce. Further, proposed ridge estimators also take into account the sample size and number of explanatory variables which makes our proposal more contributory and meaningful. Therefore, we believe that our approach is a more problem-sensitive form of regularization, which provides new insights in adapting regularization strength in different data environments.

In current study, we refined the ridge estimator introduced by Karaibrahimoğlu et al. [26] by exponentiating it to the ratio of two extreme eigen values raised to the power 1/2 and 1/2p, respectively and multiplied it by the sample size (n). Specifically, the two proposed ridge estimators take the following forms,


$$k_{NEW1}=n{\left(\frac{\sqrt{5}*{\hat{\sigma }}^{2}}{\lambda_{\max}\sum_{i=1}^{p}{\hat{\alpha }}_{i}^{2}}\right)}^{{\left(\frac{\lambda_{\min}}{\lambda_{\max}}\right)}^{\frac{\text{1}}{2p}}}$$ (28)

$$k_{NEW2}=n{\left(\frac{\sqrt{5}*{\hat{\sigma }}^{2}}{\lambda_{\max}\sum_{i=1}^{p}{\hat{\alpha }}_{i}^{2}}\right)}^{{\left(\frac{\lambda_{\min}}{\lambda_{\max}}\right)}^{\frac{\text{1}}{\text{2}}}}$$ (29)

with usual definitions of ${\hat{\sigma }}^{\text{2}}$, ${\hat{\alpha }}_{i}$, $\lambda_{min}$ and $\lambda_{max}$ already provided in previous sections.

### 2.3 Simulation scheme

The performance of the proposed estimators, along with some existing ridge estimators, is assessed through Monte Carlo simulations under different scenarios. We compared the performance of different ridge estimators for different levels of multicollinearity (ρ=0.85,0.90,0.95,0.99), number of explanatory variables (p=4,6,8), error variance (σ=2,3) and sample sizes (n=30,50,100,200,300).

The simulation scheme is explained as under:

1. Generate explanatory variables using multivariate normal distribution with mean vector as zero and covariance matrix that ensures required level of correlation (ρ). For this we have used *mvtnorm* [29] add-on package in R-language [30].
  Repeat step-2 to step-4 for N=10000 times (number of Monte Carlo replications).
2. Generate random error term ${(\mu }_{j})$ from normal distribution with mean 0 and variance $\sigma^{\text{2}}$.
3. Compute values of response variable as:
  $$Y_{j}=\beta_{0}+\beta_{1}X_{j1}+\beta_{2}X_{j2}+\cdots +\beta_{p}X_{jp}+\mu_{j}$$ (30)
  for this we have set $\beta_{\text{0}}=\text{0}$ while slope coefficients are chosen such that $\beta^{\prime }\beta =\text{1}$.
4. Estimate the parameters using OLS and RR estimators using procedures given in Equations (9) and (10).
5. Finally, estimated mean square error is computed for each choice of ridge parameter as follow:

$$EMSE=\frac{\text{1}}{N}\sum_{m=1}^{N}{\left({\hat{\beta }}_{m}-\beta \right)}^{\prime }\left({\hat{\beta }}_{m}-\beta \right)$$ (31)

Estimated mean square error (EMSE) is commonly used performance indicator in studies focusing on comparison of estimation strategies [1,6,26,31–34].

## 3. Results and discussion

The results from Monte Carlo simulations for various combinations of ρ,p, and σ for different sample sizes are presented in this section.

Table 1 shows the comparison of different ridge estimators in terms of EMSE for p=4 and σ=2 with varying levels of multicollinearity and different sample sizes. The results indicate that our proposed ridge penalty performs better, as it has lower EMSE compared to other ridge penalties. The proposed ridge estimator ($k_{New\text{2}}$) has the lowest value of EMSE for all sample sizes and for all levels of multicollinearity.

**Table 1:** Estimated MSE for different values of ρ with (p=4,σ=2).

*n*	OLS	$k_{HK}$	$k_{HKB}$	$k_{LW}$	$k_{HSL}$	$k_{KAM}$	$k_{KGM}$	$k_{KMED}$	$k_{KS}$	$k_{KMS}$	$k_{KAG}$	$k_{SCI}$	$k_{new1}$	$k_{new2}$
ρ=0.85														
30	5.468	1.883	1.722	4.591	0.986	0.600	0.798	0.237	3.252	0.386	5.396	0.729	1.354	**0.141**
50	2.915	1.036	0.944	2.736	0.792	0.431	0.483	0.220	1.814	0.462	2.895	0.667	0.789	**0.093**
100	1.382	0.545	0.508	1.362	0.600	0.308	0.274	0.202	0.961	0.596	1.379	0.558	0.430	**0.058**
200	0.671	0.323	0.298	0.669	0.411	0.243	0.163	0.187	0.529	0.691	0.671	0.399	0.244	**0.039**
300	0.451	0.238	0.226	0.450	0.317	0.220	0.121	0.181	0.378	0.724	0.451	0.311	0.180	**0.032**
ρ=0.90														
30	9.044	3.038	2.739	6.878	0.943	0.710	1.106	0.199	5.224	0.407	8.918	0.671	1.488	**0.108**
50	4.782	1.603	1.456	4.339	0.837	0.506	0.676	0.175	2.817	0.371	4.745	0.695	0.882	**0.075**
100	2.252	0.817	0.752	2.200	0.700	0.345	0.385	0.156	1.450	0.514	2.245	0.643	0.473	**0.049**
200	1.092	0.445	0.421	1.086	0.526	0.238	0.217	0.138	0.788	0.634	1.091	0.509	0.263	**0.035**
300	0.714	0.315	0.306	0.713	0.419	0.209	0.160	0.137	0.552	0.688	0.714	0.411	0.188	**0.030**
ρ=0.95														
30	18.52	5.980	5.297	11.27	0.770	0.912	1.821	0.172	10.27	0.612	18.25	0.529	1.444	**0.079**
50	10.27	3.293	2.923	8.448	0.777	0.713	1.186	0.133	5.724	0.279	10.19	0.621	0.889	**0.057**
100	4.822	1.572	1.425	4.592	0.760	0.466	0.664	0.108	2.815	0.353	4.805	0.688	0.465	**0.042**
200	2.364	0.843	0.777	2.336	0.681	0.315	0.390	0.099	1.507	0.506	2.361	0.653	0.257	**0.033**
300	1.515	0.564	0.531	1.507	0.585	0.237	0.270	0.093	1.022	0.590	1.514	0.571	0.177	**0.030**
ρ=0.99														
30	99.63	30.10	27.67	23.07	0.301	1.049	6.094	0.397	54.02	6.627	98.06	0.193	0.760	**0.061**
50	53.70	16.69	14.63	25.74	0.352	1.026	3.902	0.248	28.79	1.165	53.20	0.262	0.476	**0.049**
100	25.09	7.928	6.825	19.97	0.452	0.913	2.213	0.124	13.53	0.187	24.98	0.393	0.236	**0.039**
200	12.29	3.902	3.367	11.56	0.586	0.699	1.297	0.078	6.734	0.179	12.26	0.550	0.119	**0.035**
300	8.214	2.659	2.322	7.995	0.661	0.580	0.979	0.067	4.612	0.241	8.203	0.636	0.083	**0.034**

Values in bold represent the minimum values in each row.

Similarly, results in Table 2 (for p=4,σ=3) also show the superiority of $k_{New\text{2}}$, as it has a lower EMSE for all cases irrespective of the levels of multicollinearity and sample size. Results given in Table 3 (for p=6,σ=2), also indicate that the proposed ridge estimator consistently performs better than existing ridge choices, as it has substantially lower value of EMSE in all cases considered with different levels of multicollinearity and sample size.

**Table 2:** Estimated MSE for different values of ρ with (p=4,σ=3).

*n*	OLS	$k_{HK}$	$k_{HKB}$	$k_{LW}$	$k_{HSL}$	$k_{KAM}$	$k_{KGM}$	$k_{KMED}$	$k_{KS}$	$k_{KMS}$	$k_{KAG}$	$k_{SCI}$	$k_{new1}$	$k_{new2}$
ρ=0.85														
30	12.34	4.166	3.679	8.950	1.231	0.886	1.442	0.369	7.284	0.556	12.16	1.358	2.870	**0.275**
50	6.706	2.282	1.989	5.909	1.077	0.665	0.910	0.320	4.131	0.507	6.650	1.342	1.711	**0.179**
100	3.179	1.146	1.023	3.078	0.853	0.450	0.521	0.277	2.173	0.665	3.168	1.155	0.901	**0.103**
200	1.498	0.591	0.541	1.487	0.631	0.323	0.298	0.265	1.144	0.796	1.496	0.833	0.474	**0.063**
300	0.999	0.416	0.394	0.996	0.519	0.277	0.219	0.252	0.810	0.840	0.998	0.657	0.338	**0.049**
ρ=0.90														
30	19.65	6.289	5.653	12.44	1.125	1.014	1.980	0.313	11.19	0.641	19.35	1.258	3.170	**0.209**
50	10.62	3.526	3.042	8.767	1.021	0.785	1.258	0.263	6.227	0.429	10.53	1.331	1.886	**0.137**
100	5.104	1.765	1.525	4.854	0.880	0.540	0.718	0.214	3.238	0.557	5.085	1.287	0.989	**0.081**
200	2.405	0.851	0.775	2.375	0.716	0.351	0.395	0.199	1.687	0.722	2.401	1.029	0.511	**0.052**
300	1.614	0.613	0.568	1.605	0.628	0.293	0.298	0.191	1.212	0.788	1.613	0.863	0.367	**0.042**
ρ=0.95														
30	41.45	13.22	11.48	19.08	0.817	1.155	3.263	0.268	22.77	1.308	40.79	0.959	3.175	**0.141**
50	22.83	7.083	6.204	15.86	0.797	0.947	2.057	0.201	12.64	0.389	22.62	1.127	1.914	**0.095**
100	10.82	3.462	3.045	9.770	0.792	0.722	1.217	0.151	6.303	0.380	10.78	1.311	0.998	**0.060**
200	5.234	1.714	1.528	5.098	0.769	0.491	0.704	0.123	3.274	0.550	5.224	1.271	0.517	**0.043**
300	3.486	1.192	1.076	3.446	0.743	0.395	0.528	0.114	2.314	0.650	3.482	1.176	0.364	**0.037**
ρ=0.99														
30	222.8	68.10	61.13	29.94	0.295	0.950	10.53	0.436	120.2	16.49	219.3	0.346	1.719	**0.095**
50	122.1	38.92	32.98	37.37	0.299	0.941	6.890	0.282	65.48	3.182	120.9	0.443	1.037	**0.070**
100	56.74	17.15	15.14	36.27	0.330	0.957	3.912	0.149	30.47	0.375	56.48	0.673	0.515	**0.051**
200	27.81	8.645	7.466	24.40	0.432	0.911	2.359	0.088	15.28	0.195	27.75	0.984	0.265	**0.041**
300	18.40	5.918	5.016	17.34	0.516	0.836	1.776	0.068	10.31	0.249	18.37	1.161	0.181	**0.037**

Values in bold represent the minimum values in each row.

**Table 3:** Estimated MSE for different values of ρ with (p=6,σ=2).

*n*	OLS	$k_{HK}$	$k_{HKB}$	$k_{LW}$	$k_{HSL}$	$k_{KAM}$	$k_{KGM}$	$k_{KMED}$	$k_{KS}$	$k_{KMS}$	$k_{KAG}$	$k_{SCI}$	$k_{new1}$	$k_{new2}$
ρ=0.85														
30	9.872	3.571	2.666	7.716	1.530	0.549	0.923	0.191	5.864	0.342	9.792	0.873	3.817	**0.162**
50	5.119	1.877	1.432	4.701	1.267	0.412	0.550	0.179	3.123	0.343	5.097	0.867	2.186	**0.106**
100	2.340	0.890	0.723	2.294	0.928	0.297	0.288	0.165	1.546	0.504	2.336	0.734	1.100	**0.061**
200	1.127	0.457	0.410	1.122	0.635	0.250	0.160	0.155	0.833	0.629	1.127	0.547	0.585	**0.038**
300	0.742	0.314	0.304	0.741	0.487	0.229	0.114	0.152	0.586	0.681	0.742	0.434	0.409	**0.029**
ρ=0.90														
30	15.65	5.709	4.076	10.98	1.487	0.667	1.320	0.153	9.091	0.419	15.52	0.811	4.798	**0.116**
50	8.225	2.982	2.179	7.213	1.343	0.452	0.777	0.135	4.842	0.267	8.188	0.873	2.797	**0.078**
100	3.762	1.390	1.076	3.647	1.090	0.317	0.424	0.120	2.338	0.404	3.755	0.833	1.416	**0.047**
200	1.811	0.683	0.583	1.798	0.800	0.236	0.224	0.108	1.236	0.557	1.810	0.669	0.744	**0.030**
300	1.187	0.474	0.424	1.183	0.644	0.209	0.159	0.101	0.864	0.627	1.186	0.559	0.520	**0.024**
ρ=0.95														
30	33.99	12.07	8.589	18.32	1.270	0.994	2.428	0.141	19.46	1.013	33.71	0.621	6.574	**0.074**
50	17.56	6.371	4.424	13.56	1.260	0.666	1.394	0.104	10.07	0.246	17.48	0.750	3.786	**0.051**
100	7.982	2.876	2.072	7.484	1.218	0.408	0.741	0.081	4.662	0.243	7.964	0.865	1.910	**0.033**
200	3.873	1.394	1.079	3.812	1.052	0.263	0.407	0.069	2.388	0.399	3.869	0.836	1.002	**0.023**
300	2.533	0.955	0.753	2.515	0.902	0.212	0.283	0.062	1.636	0.496	2.531	0.749	0.689	**0.020**
ρ=0.99														
30	181.1	65.21	45.01	36.82	0.504	1.731	9.453	0.420	102.6	12.58	179.5	0.214	8.132	**0.043**
50	93.69	33.44	23.07	39.79	0.587	1.466	5.565	0.236	52.78	2.239	93.23	0.295	4.849	**0.032**
100	42.45	15.07	10.24	31.68	0.784	1.050	2.884	0.117	23.74	0.210	42.35	0.464	2.395	**0.024**
200	20.44	7.217	4.989	18.87	1.019	0.674	1.589	0.068	11.52	0.094	20.41	0.681	1.218	**0.021**
300	13.36	4.735	3.319	12.90	1.112	0.502	1.118	0.051	7.634	0.139	13.35	0.792	0.820	**0.019**

Values in bold represent the minimum values in each row.

From results given in Tables 4–6, it is evident that proposed ridge estimator ($k_{New\text{2}})$ performs better than all competing ridge estimators, except for sample size 30 and ρ=0.85 and ρ=0.90 (only in case of p=8). In fact, in such cases as the level of multicollinearity gets severer, the superiority of proposed estimator gets more profound. For n=30 and ρ=0.85, the estimator based on median ($k_{KMED}$) proposed by Kibria (2003), has lower EMSE values.

**Table 4:** Estimated MSE for different values of ρ with (p=6,σ=3).

*n*	OLS	$k_{HK}$	$k_{HKB}$	$k_{LW}$	$k_{HSL}$	$k_{KAM}$	$k_{KGM}$	$k_{KMED}$	$k_{KS}$	$k_{KMS}$	$k_{KAG}$	$k_{SCI}$	$k_{new1}$	$k_{new2}$
ρ=0.85														
30	21.92	8.054	5.710	14.48	2.101	0.869	1.776	0.292	12.94	0.597	21.73	1.593	8.326	0.341
50	11.48	4.239	3.030	9.742	1.720	0.623	1.059	0.256	6.986	0.392	11.43	1.623	4.745	**0.215**
100	5.210	1.911	1.443	4.995	1.324	0.423	0.570	0.232	3.387	0.547	5.199	1.450	2.332	**0.119**
200	2.551	0.952	0.776	2.524	0.982	0.309	0.310	0.217	1.817	0.712	2.548	1.118	1.215	**0.067**
300	1.645	0.657	0.542	1.638	0.787	0.271	0.216	0.220	1.247	0.785	1.644	0.891	0.821	**0.049**
ρ=0.90														
30	35.41	12.88	8.952	19.78	1.891	1.042	2.509	0.245	20.48	0.932	35.10	1.421	10.66	**0.244**
50	18.28	6.596	4.642	14.32	1.661	0.766	1.535	0.197	10.72	0.340	18.20	1.588	6.086	**0.156**
100	8.483	3.057	2.237	7.939	1.404	0.485	0.820	0.165	5.229	0.422	8.464	1.593	3.045	**0.086**
200	4.070	1.479	1.133	4.003	1.128	0.323	0.431	0.152	2.697	0.612	4.066	1.332	1.547	**0.051**
300	2.675	0.998	0.792	2.656	0.959	0.274	0.314	0.151	1.870	0.706	2.674	1.128	1.058	**0.038**
ρ=0.95														
30	77.53	27.77	19.39	30.37	1.447	1.465	4.707	0.208	44.54	2.748	76.87	1.063	14.82	**0.148**
50	39.85	14.38	9.853	25.32	1.357	1.092	2.794	0.153	22.82	0.496	39.65	1.298	8.557	**0.095**
100	18.19	6.428	4.584	15.93	1.315	0.685	1.473	0.107	10.68	0.257	18.15	1.581	4.261	**0.055**
200	8.649	3.084	2.216	8.355	1.252	0.429	0.792	0.085	5.274	0.412	8.640	1.585	2.122	**0.035**
300	5.756	2.088	1.531	5.668	1.165	0.331	0.560	0.083	3.656	0.527	5.753	1.463	1.452	**0.028**
ρ=0.99														
30	403.1	144.3	99.77	47.80	0.484	1.786	17.74	0.451	228.4	33.52	399.6	0.343	18.20	**0.072**
50	210.7	74.46	50.80	57.51	0.479	1.592	10.24	0.267	118.1	6.094	209.6	0.461	10.64	**0.051**
100	96.32	33.91	23.14	56.60	0.568	1.395	5.571	0.141	53.95	0.570	96.09	0.750	5.418	**0.033**
200	45.90	15.97	11.21	38.90	0.754	1.095	3.124	0.074	26.07	0.122	45.84	1.136	2.718	**0.025**
300	30.11	10.67	7.336	27.88	0.893	0.864	2.192	0.054	17.21	0.136	30.08	1.363	1.816	**0.022**

Values in bold represent the minimum values in each row.

**Table 5:** Estimated MSE for different values of ρ with (p=8,σ=2).

*n*	OLS	$k_{HK}$	$k_{HKB}$	$k_{LW}$	$k_{HSL}$	$k_{KAM}$	$k_{KGM}$	$k_{KMED}$	$k_{KS}$	$k_{KMS}$	$k_{KAG}$	$k_{SCI}$	$k_{new1}$	$k_{new2}$
ρ=0.85														
30	15.06	5.916	3.729	11.02	2.083	0.512	1.065	0.165	9.029	0.398	14.98	1.004	7.519	0.186
50	7.559	2.977	1.918	6.770	1.703	0.386	0.606	0.155	4.625	0.276	7.536	1.013	4.088	**0.120**
100	3.293	1.327	0.925	3.214	1.251	0.297	0.308	0.143	2.137	0.434	3.289	0.898	1.945	**0.068**
200	1.601	0.657	0.517	1.591	0.849	0.256	0.164	0.134	1.143	0.577	1.600	0.673	1.008	**0.039**
300	1.033	0.422	0.369	1.030	0.644	0.239	0.116	0.131	0.782	0.643	1.033	0.534	0.677	**0.029**
ρ=0.90														
30	24.16	9.152	5.811	15.46	2.049	0.627	1.565	0.134	14.32	0.610	24.02	0.916	10.28	**0.133**
50	12.11	4.693	2.971	10.27	1.843	0.427	0.892	0.112	7.256	0.224	12.07	1.021	5.693	**0.087**
100	5.446	2.134	1.419	5.236	1.489	0.295	0.467	0.098	3.386	0.321	5.439	1.013	2.766	**0.049**
200	2.552	1.038	0.734	2.528	1.067	0.233	0.236	0.089	1.695	0.493	2.551	0.819	1.377	**0.029**
300	1.641	0.660	0.515	1.634	0.839	0.207	0.164	0.087	1.150	0.577	1.640	0.675	0.924	**0.023**
ρ=0.95														
30	51.38	19.71	12.03	24.81	1.748	0.927	2.895	0.120	30.22	1.670	51.09	0.688	16.18	**0.079**
50	26.43	9.921	6.196	19.27	1.784	0.634	1.717	0.085	15.57	0.307	26.35	0.861	9.266	**0.051**
100	11.59	4.497	2.796	10.69	1.712	0.364	0.843	0.066	6.919	0.175	11.57	1.035	4.424	**0.031**
200	5.495	2.115	1.402	5.387	1.439	0.243	0.452	0.052	3.381	0.322	5.492	1.020	2.216	**0.020**
300	3.580	1.399	0.961	3.549	1.219	0.199	0.315	0.045	2.267	0.422	3.578	0.921	1.492	**0.017**
ρ=0.99														
30	273.5	105.4	63.24	50.30	0.681	2.142	12.26	0.382	159.8	20.37	271.9	0.224	33.00	**0.035**
50	138.4	51.68	31.48	53.78	0.817	1.590	6.850	0.201	80.45	3.439	138.0	0.313	19.29	**0.024**
100	62.01	23.23	14.07	43.48	1.129	1.033	3.514	0.105	35.91	0.329	61.91	0.513	9.475	**0.018**
200	29.10	11.05	6.729	26.37	1.464	0.626	1.882	0.056	16.97	0.069	29.08	0.779	4.745	**0.014**
300	19.09	7.391	4.466	18.26	1.600	0.450	1.310	0.044	11.21	0.087	19.09	0.930	3.186	**0.013**

Values in bold represent the minimum values in each row.

**Table 6:** Estimated MSE for different values of ρ with (p=8,σ=3).

*n*	OLS	$k_{HK}$	$k_{HKB}$	$k_{LW}$	$k_{HSL}$	$k_{KAM}$	$k_{KGM}$	$k_{KMED}$	$k_{KS}$	$k_{KMS}$	$k_{KAG}$	$k_{SCI}$	$k_{new1}$	$k_{new2}$
ρ=0.85														
30	33.72	13.22	8.075	20.30	2.896	0.837	2.146	0.250	20.18	0.888	33.51	1.733	16.55	0.400
50	17.03	6.574	4.143	13.83	2.387	0.572	1.217	0.221	10.41	0.337	16.97	1.854	9.094	**0.216**
100	7.596	2.994	1.940	7.204	1.821	0.388	0.624	0.200	4.862	0.449	7.585	1.730	4.317	**0.139**
200	3.553	1.399	0.984	3.507	1.318	0.305	0.334	0.192	2.444	0.646	3.550	1.344	2.121	**0.076**
300	2.332	0.917	0.693	2.319	1.059	0.277	0.234	0.189	1.695	0.730	2.331	1.097	1.435	**0.054**
ρ=0.90														
30	54.84	21.10	13.01	27.65	2.648	1.060	3.205	0.200	32.55	1.619	54.52	1.552	23.22	0.285
50	27.03	10.44	6.375	19.97	2.285	0.690	1.775	0.183	16.12	0.345	26.94	1.772	12.48	**0.180**
100	12.02	4.637	2.920	11.07	1.924	0.451	0.907	0.138	7.401	0.332	12.01	1.850	5.933	**0.098**
200	5.725	2.215	1.465	5.609	1.537	0.303	0.476	0.127	3.714	0.528	5.722	1.594	2.954	**0.054**
300	3.843	1.506	1.037	3.808	1.306	0.254	0.339	0.122	2.612	0.630	3.842	1.373	2.034	**0.040**
ρ=0.95														
30	115.1	43.71	26.48	39.85	2.004	1.531	5.787	0.176	67.33	4.621	114.5	1.094	35.83	**0.162**
50	57.84	22.46	13.45	34.41	1.854	1.073	3.379	0.123	34.03	0.748	57.65	1.403	20.35	**0.102**
100	26.20	10.06	6.088	22.21	1.852	0.632	1.725	0.084	15.57	0.197	26.16	1.794	9.803	**0.057**
200	12.26	4.725	2.959	11.75	1.772	0.392	0.917	0.068	7.494	0.315	12.25	1.892	4.832	**0.033**
300	8.078	3.105	1.993	7.925	1.624	0.303	0.639	0.063	5.072	0.433	8.074	1.758	3.237	**0.025**
ρ=0.99														
30	622.8	239.3	143.5	65.71	0.677	2.492	24.79	0.466	364.6	58.02	619.2	0.342	74.55	**0.065**
50	308.3	117.9	70.09	78.96	0.668	2.074	13.74	0.260	179.4	10.18	307.3	0.474	43.10	**0.043**
100	139.8	53.45	32.09	75.82	0.802	1.626	7.151	0.129	81.71	1.071	139.6	0.780	21.45	**0.027**
200	66.26	25.19	14.97	54.02	1.082	1.075	3.749	0.064	38.66	0.118	66.21	1.228	10.59	**0.018**
300	42.56	16.05	9.699	38.69	1.273	0.779	2.563	0.044	24.90	0.089	42.54	1.498	6.972	**0.016**

Values in bold represent the minimum values in each row.

It is worth to mention that in cases where $k_{KMED}$ performs better among all ridge estimators, our proposed estimator ($k_{New\text{2}})$ is second-best choice. Similarly, in all those cases where newly proposed ridge estimator $\left(k_{New\text{2}}\right)$ found better than competing estimators in terms of EMSE, the Kibria’s estimator ($k_{KMED})$ emerges as the second-best choice.

**Fig 1:**
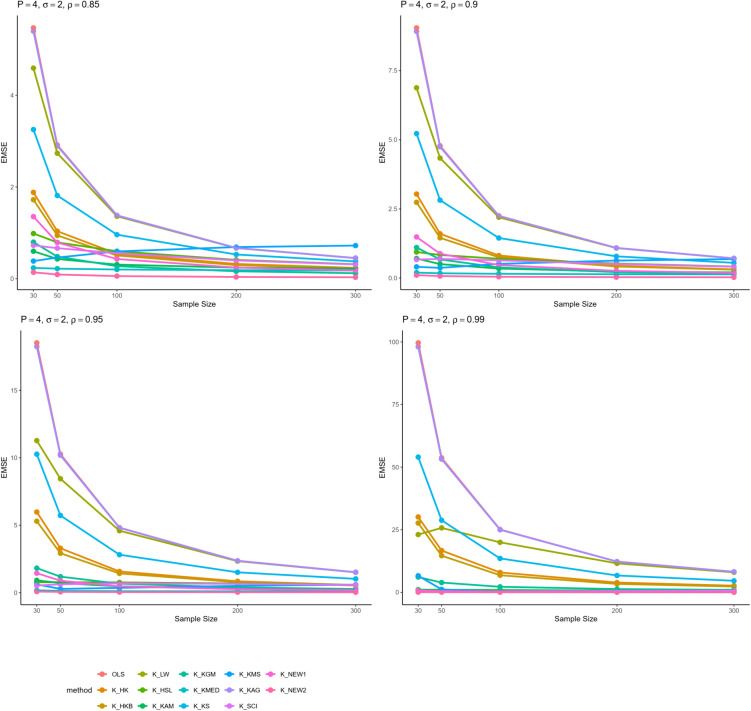
Comparison of EMSE of ridge estimators for 𝐏=4,σ=2 for different values of ρ.

**Fig 2:**
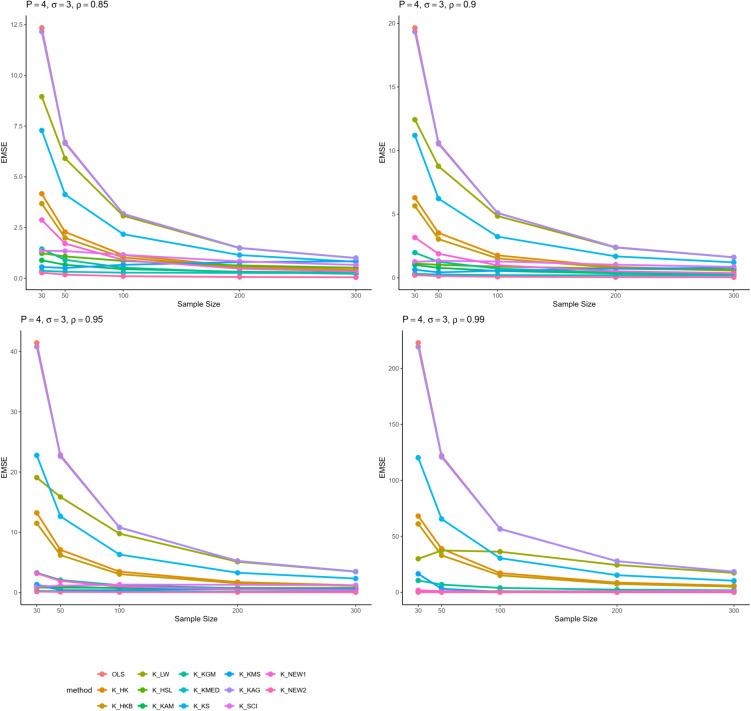
Comparison of EMSE of ridge estimators for 𝐏=4,σ=3 for different values of ρ.

**Fig 3:**
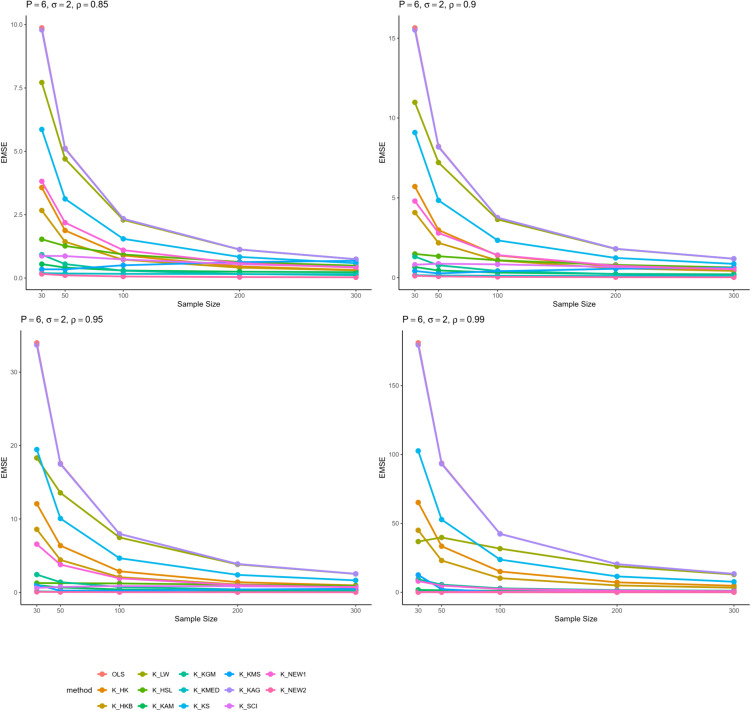
Comparison of EMSE of ridge estimators for 𝐏=6,σ=2 for different values of ρ.

**Fig 4:**
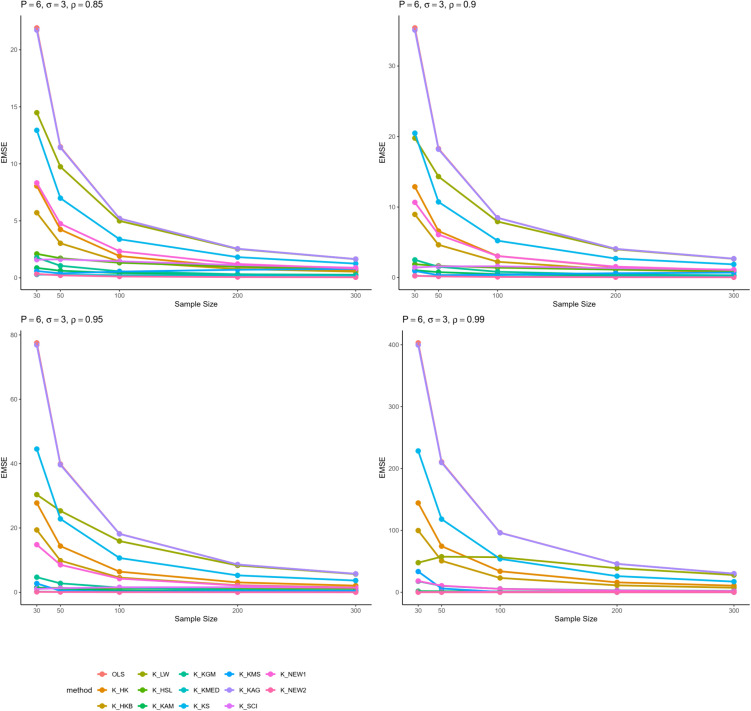
Comparison of EMSE of ridge estimators for 𝐏=6,σ=3 for different values of ρ.

**Fig 5:**
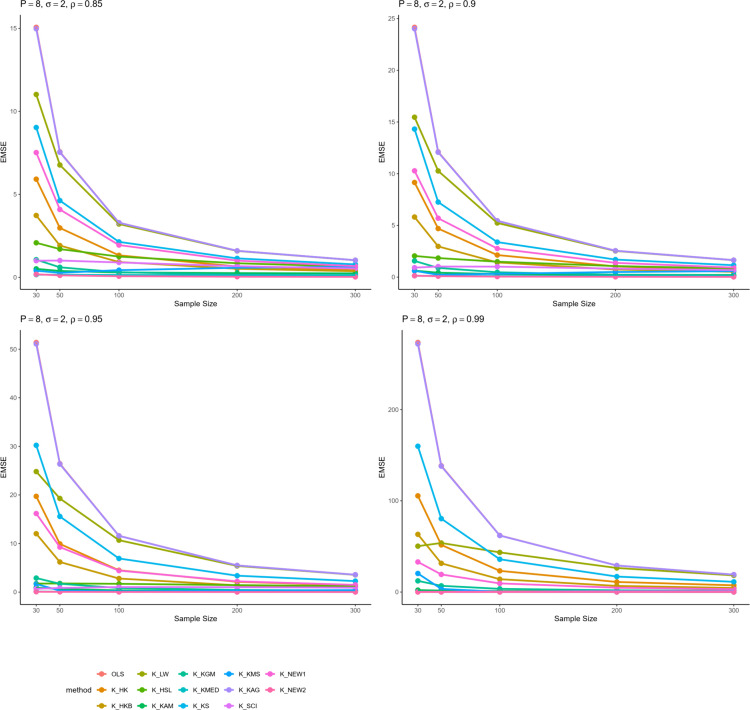
Comparison of EMSE of ridge estimators for 𝐏=8,σ=2 for different values of ρ.

**Fig 6:**
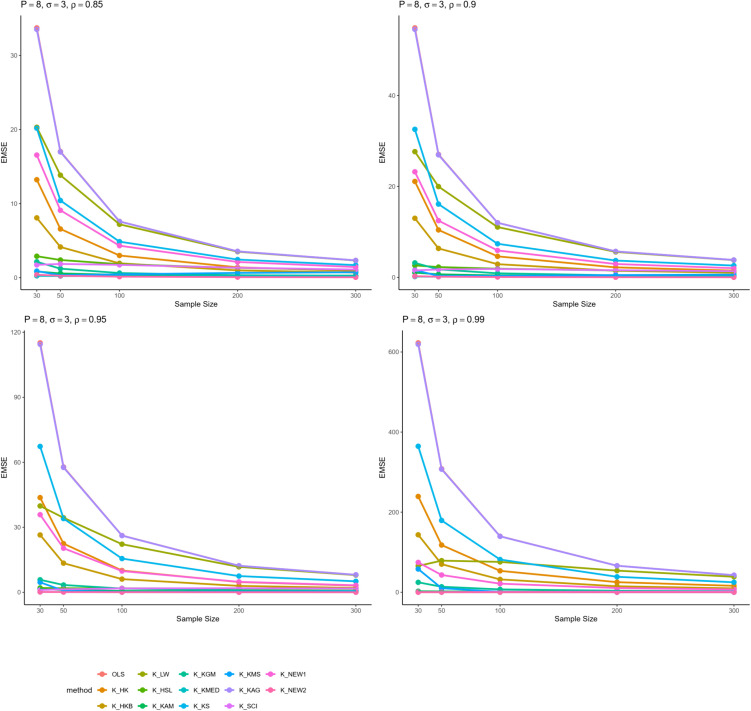
Comparison of EMSE of ridge estimators for 𝐏=8,σ=3 for different values of ρ.

From all these results, we can infer some general findings as well. For instance, all ridge estimators perform better than OLS estimation in general linear model in cases of multicollinearity. Moreover, the EMSE decreases with increasing sample size. The EMSE gets larger for stronger levels of multicollinearity which suggests a possible adverse effect. The results given in Tables 1–6 are graphically represented in Figs 1–6.

## 4. Real data applications

In addition to the simulation study, the performance of proposed and existing ridge estimators has also been evaluated on two real-life multicollinear datasets. Owing to absence of repeated sampling, for real-life applications, mean square error is estimated using empirical formulas given in Equations 14 and 15. We have also compared the amount of shrinkage achieved by different ridge parameters in comparison to OLS estimation. The comparison in the amount of overall shrinkage among different ridge estimators is assessed through absolute shrinkage (AS) and relative shrinkage (RS) which are computed as:


$$AS={\left\|{\hat{\beta }}_{OLS}\right\|}^{2}-{\left\|{\hat{\beta }}_{Ridge}\right\|}^{2}$$ (32)

$$AS=\frac{{\left\|{\hat{\beta }}_{OLS}\right\|}^{2}-{\left\|{\hat{\beta }}_{Ridge}\right\|}^{2}}{{\left\|{\hat{\beta }}_{OLS}\right\|}^{2}}$$ (33)

The performance of different ridge estimators is also evaluated in terms of the coverage percentage of prediction intervals. The coverage percentage of prediction intervals (CPPI) is computed as:


$$CPPI=100*\sum_{j=1}^{n}I_{j}\left\{y_{j}\in \left(LL_{y_{j}},UL_{y_{j}}\right)\right\}$$ (34)

where


$$LL_{y_{j}}={\hat{y}}_{j}-t_{\left(\alpha /2,df\right)}\sqrt{{\hat{\sigma }}^{2}\left(1+x{j*}^{\prime }{\left({X^{*}}^{\prime }X^{*}\right)}^{-1}x_{j}^{*}\right)}$$ (35)

$$UL_{y_{j}}={\hat{y}}_{j}+t_{\left(\alpha /2,df\right)}\sqrt{{\hat{\sigma }}^{2}\left(1+x{j*}^{\prime }{\left({X^{*}}^{\prime }X^{*}\right)}^{-1}x_{j}^{*}\right)}$$ (36)

where, ${\hat{y}}_{j}$ and ${\hat{\sigma }}^{\text{2}}$ are estimates of the $j^{th}$ response and error variance based on a particular ridge parameter, α is the common level of significance, while $X^{*}$ and $x_{j}^{*}$ have usual definitions. The description of data and results by employing different ridge estimators are given in the following sub-sections.

### 4.1 Bodyfat data

The first real-life application is based on body fat data [35]. The body fat data consist of features that can be used to produce a predictive model for body fat percentage from Siri’s equation [36]. The same data has been used in some other studies [37–40] and it is available in add-an packages like, *gRbase* [41] and *UsingR* [42] in R-language [30]. We have taken data for individuals aged between 32–40 years. The reason for selecting this age group is that it has the most severe multicollinearity among explanatory variables, as measured by the condition number of their correlation matrix. The dataset consists of 50 observations with a response Y (body fat percentage) and 13 explanatory variables: X1 (Chest circumference in cm), X2 (Abdomen circumference in cm), X3 (Hip circumference in cm), X4 (Thigh circumference in cm), X5 (Knee circumference in cm), X6 (Ankle circumference in cm), X7 (Biceps circumference in cm), X8 (Forearm circumference in cm), X9 (Neck circumference in cm), X10 (Wrist circumference in cm), X11 (Weight in lbs), X12 (Height in inches) and X13 (Density determined from underwater weighing). The correlation matrix (Table 7), condition number (18.33), VIF (15.03, 28.84, 30.41, 15.81, 8.65, 1.74, 7.31, 2.67, 7.63, 5.25, 96.07, 4.72, 4.56) and Farrar and Glauber [43] statistic (933.26) clearly indicates that strong multicollinearity is present in the data.

**Table 7:** Correlation matrix of body fat data.

	X1	X2	X3	X4	X5	X6	X7	X8	X9	X10	X11	X12	X13
X1	1	0.9412	0.9069	0.8590	0.8091	0.4774	0.7922	0.5437	0.8114	0.7415	0.9349	0.4897	−0.7334
X2	0.9412	1	0.9505	0.9156	0.8528	0.4214	0.8016	0.5447	0.7968	0.7195	0.9521	0.5309	−0.8134
X3	0.9069	0.9505	1	0.9289	0.9076	0.4926	0.8359	0.5411	0.8088	0.7873	0.9648	0.5844	−0.7429
X4	0.8590	0.9156	0.9289	1	0.8580	0.4354	0.8756	0.5897	0.7993	0.7222	0.9102	0.4506	−0.7684
X5	0.8091	0.8528	0.9076	0.8580	1	0.5116	0.8066	0.5576	0.7570	0.7302	0.9054	0.6871	−0.6475
X6	0.4774	0.4214	0.4926	0.4354	0.5116	1	0.4899	0.3635	0.4906	0.5619	0.5350	0.5184	−0.2328
X7	0.7922	0.8016	0.8359	0.8756	0.8066	0.4899	1	0.7029	0.8514	0.7483	0.8752	0.5428	−0.6402
X8	0.5437	0.5447	0.5411	0.5897	0.5576	0.3635	0.7029	1	0.6884	0.5604	0.6484	0.4592	−0.4322
X9	0.8114	0.7968	0.8088	0.7993	0.7570	0.4906	0.8514	0.6884	1	0.8236	0.8865	0.5748	−0.5422
X10	0.7415	0.7195	0.7873	0.7222	0.7302	0.5619	0.7483	0.5604	0.8236	1	0.8311	0.5935	−0.3969
X11	0.9349	0.9521	0.9648	0.9102	0.9054	0.5350	0.8752	0.6484	0.8865	0.8311	1	0.6662	−0.7278
X12	0.4897	0.5309	0.5844	0.4506	0.6871	0.5184	0.5428	0.4592	0.5748	0.5935	0.6662	1	−0.3928
X13	−0.7334	−0.8134	−0.7429	−0.7684	−0.6475	−0.2328	−0.6402	−0.4322	−0.5422	−0.3969	−0.7278	−0.3928	1

The results by applying all competing ridge penalties are given in Table 8. These results show better performance of the proposed ridge parameter $\left(K_{New\text{2}}\right)$ as it has the lower EMSE value (0.010) than all competing choices for ridge parameter. These results reveal that ridge estimator given by Khalaf et al. [15] is the second-best choice in terms of EMSE. The proposed ridge estimator ($K_{New\text{2}})$ achieves maximum amount of shrinkage as indicated by absolute and relative shrinkage measures. The coverage percentage of prediction intervals is almost similar for all estimators ranging from 94% to 96% coverage.

**Table 8:** Estimated MSE and parameter estimates for body fat data.

	OLS	$k_{HK}$	$k_{HKB}$	$k_{LW}$	$k_{HSL}$	$k_{KAM}$	$k_{KGM}$	$k_{KMED}$	$k_{KS}$	$k_{KMS}$	$k_{KAG}$	$k_{SCI}$	$k_{New\text{1}}$	$k_{New\text{2}}$
AS	---	0.033	0.060	0.002	0.003	0.149	0.081	0.071	0.032	0.483	0.000	0.064	0.015	0.535
RS	---	0.034	0.063	0.002	0.003	0.156	0.085	0.074	0.034	0.506	0.000	0.067	0.015	0.561
EMSE	0.3938	0.1979	0.1263	0.3755	0.3679	0.0530	0.0963	0.1089	0.1992	0.0123	0.3935	0.1199	0.2875	0.0100
${\hat{\beta }}_{\text{1}}$	-0.0844	−0.0634	−0.0443	−0.0831	−0.0826	0.0083	−0.0297	−0.0366	−0.0636	0.0811	−0.0844	−0.0417	−0.0755	0.0819
${\hat{\beta }}_{\text{2}}$	0.0366	0.0578	0.0759	0.0379	0.0385	0.1165	0.0886	0.0827	0.0576	0.1340	0.0366	0.0782	0.0457	0.1270
${\hat{\beta }}_{\text{3}}$	0.0251	0.0423	0.0513	0.0264	0.0269	0.0563	0.0545	0.0533	0.0422	0.0768	0.0252	0.0521	0.0333	0.0774
${\hat{\beta }}_{\text{4}}$	0.0096	0.0193	0.0313	0.0101	0.0103	0.0725	0.0423	0.0369	0.0191	0.1070	0.0096	0.0332	0.0132	0.1036
${\hat{\beta }}_{\text{5}}$	-0.0969	−0.0951	−0.0930	−0.0968	−0.0967	−0.0786	−0.0907	−0.0919	−0.0951	0.0157	−0.0969	−0.0926	−0.0961	0.0258
${\hat{\beta }}_{\text{6}}$	0.0051	0.0038	0.0019	0.0050	0.0050	−0.0092	−0.0003	0.0008	0.0039	−0.0379	0.0050	0.0015	0.0046	−0.0354
${\hat{\beta }}_{\text{7}}$	0.0500	0.0523	0.0537	0.0502	0.0502	0.0550	0.0544	0.0541	0.0523	0.0531	0.0500	0.0539	0.0511	0.0532
${\hat{\beta }}_{\text{8}}$	-0.0367	−0.0308	−0.0259	−0.0363	−0.0362	−0.0134	−0.0224	−0.0241	−0.0309	0.0103	−0.0367	−0.0253	−0.0342	0.0130
${\hat{\beta }}_{\text{9}}$	0.0383	0.0416	0.0409	0.0386	0.0387	0.0213	0.0377	0.0395	0.0416	−0.0015	0.0383	0.0405	0.0402	0.0040
${\hat{\beta }}_{\text{1}\text{0}}$	-0.1563	−0.1544	−0.1542	−0.1561	−0.1561	−0.1600	−0.1551	−0.1545	−0.1544	−0.1046	−0.1563	−0.1543	−0.1553	−0.0847
${\hat{\beta }}_{\text{1}\text{1}}$	0.2830	0.2070	0.1530	0.2779	0.2758	0.0773	0.1221	0.1356	0.2078	0.0660	0.2829	0.1468	0.2491	0.0663
${\hat{\beta }}_{\text{1}\text{2}}$	-0.0314	−0.0191	−0.0089	−0.0306	−0.0303	0.0107	−0.0020	−0.0051	−0.0192	0.0063	−0.0314	−0.0076	−0.0261	0.0063
${\hat{\beta }}_{\text{1}\text{3}}$	-0.8828	−0.8696	−0.8520	−0.8821	−0.8819	−0.7642	−0.8338	−0.8429	−0.8698	−0.3995	−0.8828	−0.8490	−0.8779	−0.3448
CPPI	96%	96%	96%	96%	96%	96%	96%	96%	96%	94%	96%	96%	96%	94%

### 4.2 Livestock data

The second real data comparison is performed using the livestock data [32] from economic survey of Pakistan which is a yearly report covering all sectors of economy including agriculture, industry, livestock etc. The livestock data consists of 18 observations and five explanatory variables for modelling the response variable Y (production of hair measured in tons). The explanatory variables (taken in millions) are, X1 (buffalos), X2 (cattle), X3 (goats), X4 (sheep), and X5 (poultry). There exists a strong multicollinearity in the data as indicated by correlation matrix among explanatory variables (Table 9), VIF values (17605.65, 9262.50, 4150.09, 3934.14, 721.66), condition number (359.97) and value of Farrar-Glauber statistic (356.26). The same data have been used by Dar et al. [1] for comparison among different ridge penalties.

**Table 9:** Correlation matrix of livestock data.

	X1	X2	X3	X4	X5
X1	1	0.9931	0.9865	0.9866	0.9872
X2	0.9931	1	0.9611	0.9975	0.9760
X3	0.9865	0.9611	1	0.9469	0.9860
X4	0.9866	0.9975	0.9469	1	0.9589
X5	0.9872	0.9760	0.9860	0.9589	1

The parameter estimates (in standardized form) and EMSE values obtained by applying all ridge constants being compared are given in Table 10. From these results, the better performance of the proposed ridge estimator ($k_{New\text{2}}$) is evident as it has minimum EMSE (0.0019) compared to all existing ridge choices. Moreover, the $k_{New\text{1}}$ is the second-best choice as it has least EMSE after $k_{Nwe\text{2}}$. Similarly, the performance of both proposed ridge parameters achieved high amount of shrinkage in terms of absolute and relative shrinkage for livestock data. Comparison based on coverage percentage of prediction intervals clearly indicates superiority of the proposed ridge estimator ($k_{new\text{2}})$ as it shows 100% coverage.

**Table 10:** Estimated MSE and parameter estimates for livestock data.

Estimator	AS	RS	MSE	${\hat{\beta }}_{\text{1}}$	${\hat{\beta }}_{\text{2}}$	${\hat{\beta }}_{\text{3}}$	${\hat{\beta }}_{\text{4}}$	${\hat{\beta }}_{\text{5}}$	CPPI
OLS	---	----	835.8433	0.1248	0.7568	0.7657	−0.6058	−0.0450	94%
$k_{HK}$	0.508	0.409	0.9217	0.2850	0.0807	0.6305	−0.1633	0.1656	89%
$k_{HKB}$	0.502	0.404	1.2664	0.2876	0.0982	0.6320	−0.1782	0.1589	89%
$k_{LW}$	0.243	0.196	149.6802	0.2624	0.4928	0.6819	−0.4702	0.0294	89%
$k_{HSL}$	0.407	0.328	21.2906	0.2974	0.2776	0.6471	−0.3218	0.0969	89%
$k_{KAM}$	0.520	0.418	0.5444	0.2787	0.0457	0.6264	−0.1324	0.1804	89%
$k_{KGM}$	0.512	0.412	0.7732	0.2834	0.0704	0.6295	−0.1543	0.1697	89%
$k_{KMED}$	0.508	0.409	0.9217	0.2850	0.0807	0.6305	−0.1633	0.1656	89%
$k_{KS}$	0.508	0.409	0.9237	0.2851	0.0809	0.6305	−0.1634	0.1655	89%
$k_{KMS}$	0.620	0.499	0.1148	0.2339	0.0230	0.5102	−0.0358	0.2647	94%
$k_{KAG}$	0.024	0.019	716.0081	0.1426	0.7323	0.7558	−0.5956	−0.0385	94%
$k_{SCI}$	0.523	0.421	0.4906	0.2766	0.0364	0.6247	−0.1238	0.1849	89%
$k_{New\text{1}}$	0.697	0.561	0.0365	0.2166	0.0695	0.4149	0.0187	0.2699	94%
$k_{New\text{2}}$	0.872	0.702	**0.0019**	0.1675	0.1520	0.1860	0.1446	0.1751	100%

## 5. Conclusions

To tackle the problem of multicollinearity in the general linear model, the present study suggests two new ridge penalties to be used in ridge regression. The proposed ridge estimator is compared with some historical as well as recent existing ridge penalties. Based on EMSE as performance metric, the Monte Carlo simulation indicated the superiority of the proposed strategy for different sample sizes under various scenarios (accounting for level of multicollinearity, number of explanatory variables and error variance) in most cases. The proposed choices for ridge constant, gets more superior than competitors with the increasing severity of multicollinearity among regressors. The simulation results were further supported by two real-life applications where the proposed estimator showed better performance as well. The results from real data application indicate that proposed ridge estimators perform much better than existing choice in case of moderate as well as strong multicollinearity. Based on our results, we recommend use of the proposed ridge estimator to tackle with the problem of multicollinearity or ill-conditioned data through ridge regression approach. The findings of the study can help practitioners to handle the adverse effects of strong multicollinearity in a more effective way.
